# Efficacy of acupuncture for chronic low back pain: protocol for a randomized controlled trial

**DOI:** 10.1186/1745-6215-9-10

**Published:** 2008-02-28

**Authors:** Daniel C Cherkin, Karen J Sherman, Charissa J Hogeboom, Janet H Erro, William E Barlow, Richard A Deyo, Andrew L Avins

**Affiliations:** 1 Center for Health Studies, Group Health Cooperative, Seattle, USA; 2 Cancer Research and Biostatistics, Seattle, USA; 3 School of Medicine, Oregon Health and Science University, Portland, USA; 4 Division of Research, Northern California Kaiser Permanente, Oakland, USA

## Abstract

**Background:**

Chronic back pain is a major public health problem and the primary reason patients seek acupuncture treatment. Therefore, an objective assessment of acupuncture efficacy is critical for making informed decisions about its appropriate role for patients with this common condition. This study addresses methodological shortcomings that have plagued previous studies evaluating acupuncture for chronic low back pain.

**Methods and Design:**

A total of 640 participants (160 in each of four arms) between the ages of 18 and 70 years of age who have low back pain lasting at least 3 months will be recruited from integrated health care delivery systems in Seattle and Oakland. They will be randomized to one of two forms of Traditional Chinese Medical (TCM) acupuncture needling (individualized or standardized), a "control" group (simulated acupuncture), or to continued usual medical care. Ten treatments will be provided over 7 weeks. Study participants and the "Diagnostician" acupuncturists who evaluate participants and propose individualized treatments will be masked to the acupuncture treatment actually assigned each participant. The "Therapist" acupuncturists providing the treatments will not be masked but will have limited verbal interaction with participants. The primary outcomes, standard measures of dysfunction and bothersomeness of low back pain, will be assessed at baseline, and after 8, 26, and 52 weeks by telephone interviewers masked to treatment assignment. General health status, satisfaction with back care, days of back-related disability, and use and costs of healthcare services for back pain will also be measured. The primary analysis comparing outcomes by randomized treatment assignment will be analysis of covariance adjusted for baseline value. For both primary outcome measures, this trial will have 99% power to detect the presence of a minimal clinically significant difference among all four treatment groups and over 80% power for most pairwise comparisons. Secondary analyses will compare the proportions of participants in each group that improve by a clinically meaningful amount.

**Conclusion:**

Results of this trial will help clarify the value of acupuncture needling as a treatment for chronic low back pain.

**Trial registration:**

Clinical Trials.gov NCT00065585.

## Background

Back pain is one of the most important health problems in developed countries. More than 50% of adults are bothered by back pain each year [1] and 70% to 80% of adults are afflicted by it at some time in their lives [2]. Back symptoms are the leading cause of visits to orthopedic surgeons and neurosurgeons and the second leading symptomatic reason for visits to all physicians [3]. Back pain is the most costly ailment of working-age adults [4]. An estimated $25 billion are spent on personal medical care for back pain every year and compensation and lost productivity costs are much higher [5].

Back pain patients are often dissatisfied with conventional medical care [6], especially in comparison to care provided by non-MD's including CAM practitioners [7-10]. Back pain is the leading primary reason for visits to licensed acupuncturists, representing about 1 in 7 visits [11], and acupuncturists consider it to be one of the conditions for which acupuncture is most effective [12].

Despite numerous published randomized trials evaluating acupuncture as a treatment for chronic low back pain, the efficacy and effectiveness of acupuncture for this common problem remains unclear. The 1997 NIH Consensus Development Panel on Acupuncture noted "many of these studies provide equivocal results because of design, sample size, and other factors. The issue is further complicated by inherent difficulties in the use of appropriate controls, such as placebo and sham acupuncture groups" [13]. Other reviews published before the study protocol described in this manuscript was proposed (2002) also noted the poor quality of research in this area and urged that scientifically rigorous studies be conducted [14-16]. This study was designed to avoid the methodological shortcomings that have plagued previous studies, including inadequate acupuncture treatments, very small sample sizes, failure to include long-term outcome measures and high rates of loss to follow-up.

Our primary aims are to answer the following questions:

1) Is acupuncture needling more effective than usual medical care for reducing dysfunction or symptom bothersomeness due to chronic low back pain?

We hypothesize that acupuncture needling (both standardized and individualized) will be more effective than usual medical care.

2) Is acupuncture needling more effective that non-insertive simulated acupuncture for reducing dysfunction or symptom bothersomeness due to chronic low back pain?

We hypothesize that standardized acupuncture needling will be more effective than non-insertive simulated needling.

3) Is individualized acupuncture needling more effective than standardized acupuncture needling for reducing dysfunction or symptom bothersomeness due to chronic low back pain?

We hypothesize that individualized acupuncture needling will be more effective than standardized acupuncture needling.

Our secondary aims are to compare the effects of these four treatments on general health status, patient satisfaction with care, days of restricted activity due to back problems, and costs and utilization of services.

By addressing the methodological shortcomings of previous studies, this trial will clarify the extent to which acupuncture can diminish the effect of chronic low back pain on patient functioning and symptoms. Because chronic back pain is a major public health problem and the top reason patients seek acupuncture treatment, an unambiguous assessment is critical for making informed decisions about the most appropriate role for acupuncture in care for back pain.

## Methods and design

### Overview

**Figure 1:**
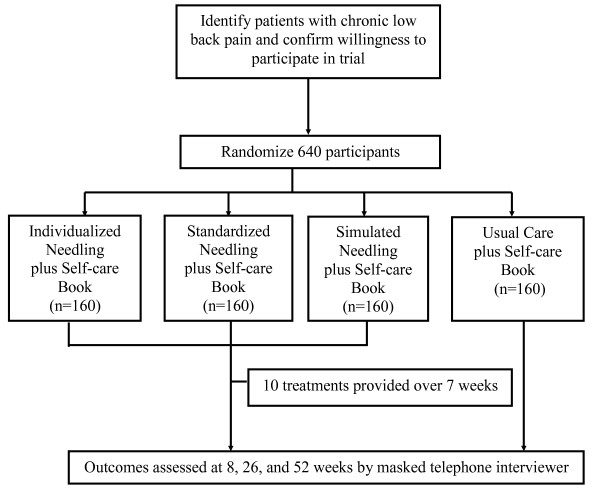
Study Design. Process of recruitment, randomization to treatment, treatment provision, and outcomes assessment.

Eligible participants at two sites will be randomized equally to one of four treatments: individualized acupuncture point stimulation, standardized acupuncture point stimulation, non-insertive simulated acupuncture point stimulation, or usual care (Figure 1). Participants in all three "acupuncture" arms will receive 10 "treatments" involving stimulation of acupuncture points over a 7-week treatment period (two treatments per week for 3 weeks, weekly treatments for 4 weeks).

The acupuncture provided will be in accordance with the principles of Traditional Chinese Medicine (TCM) in terms of point selection and needling details. Although many styles of acupuncture are used in the U.S., we selected TCM because it appears to be the most commonly practiced [11,17] is the basis of the national certification exam for non-physician acupuncturists, and is the foundation of non-physician acupuncturist training in the states in which this trial is being conducted. The treatments will be provided by licensed acupuncturists, the group that provides the vast majority of acupuncture treatments in the United States.

In addition to the assigned treatments, all participants will receive a high-quality book about self-management of back pain and will retain access to the health care services to which they are entitled by their insurance coverage.

Participants will be followed for a one-year period following randomization and primary and secondary outcomes will be assessed by telephone interviewers masked to treatment assignment after 8, 26, and 52 weeks. The primary outcome will be dysfunction due to back pain and bothersomeness of back pain. Secondary outcomes will include general health status, satisfaction with back care, days of restricted activity, and use and costs of back-related health care for the year following randomization.

Bias will be minimized by a clinical protocol designed to offer maximum possible masking in a study of a physical procedure such as acupuncture. Although full double masking is not possible, this design permits masking of the assessor of outcomes and substantial masking of participants and the acupuncturist prescribing a treatment. Participants in the three "acupuncture" groups will know only that they will receive one of several methods of stimulating acupoints.

We will perform an intention-to-treat analysis of the data, i.e., the analysis will be by randomized assignment regardless of participation in treatment sessions. This trial was preceded by a pilot trial to assess the feasibility of a full-scale trial. The pilot study demonstrated the feasibility of carrying out a full-scale trial, provided estimates of recruitment rates, follow-up rates, and sample size requirements, and helped us optimize the clinical procedures [18].

### Study population

This study will focus on patients between 18 and 70 years of age with non-radicular chronic low back pain of mechanical origin (as opposed to infectious, neoplastic, or inflammatory causes). There are many potential causes of low back pain, but in most cases, a precise pathoanatomic diagnosis is unattainable because of the weak associations among symptoms, pathoanatomic changes, and imaging results.

### Inclusion and exclusion criteria

Entry inclusion and exclusion criteria (Table 1) were developed with the goal of maximizing enrollment of appropriate participants while excluding patients who have low back pain of a specific (e.g., spinal stenosis) or complicated (e.g., due to a medical condition) nature, for whom acupuncture is contraindicated (e.g., clotting disorders), or whose medical conditions might make it difficult to receive the treatments (e.g., gross obesity or severe psychiatric conditions). These criteria are intended to exclude patients with medical conditions that might contribute to an increased risk of a severe adverse event, would not allow for fully informed consent, or might lead to misinterpretation of the outcomes (e.g., multiple sclerosis or diabetes whose neurological symptoms might interfere with pain sensation). To maximize the credibility of our simulated acupuncture treatment, we will also exclude people who have had acupuncture treatment in the past because such individuals may be more able to distinguish simulated from actual acupuncture.

**Table 1:** Inclusion and exclusion criteria

Inclusion criteria
Participant plans to continue enrollment in health plan
Between 18 and 70 years of age
At least one primary care visit for back pain within the past 3–12 months
Non-specific, uncomplicated low back pain, i.e., ICD-9 codes:
724.2 Lumbago
724.5 Backache, unspecified
724.8 Other symptoms referable to back
846.0-9 Sprains and strains, sacroiliac
847.2 Sprains and strains, lumbar
847.3 Sprains and strains, sacral
847.9 Sprains and strains, unspecified site of the back
Physician willing to have patients included in the study
Lives within 45 minutes travel time from study clinic

Exclusion criteria
Previous acupuncture for any reason
Low back pain lasting less than 3 months
Mild symptoms (less than 3 on 0 to 10 pain bothersomeness scale)
Specific diseases that could be cause of back pain (metastatic cancer, discitis, herniated disc, vertebral fracture, spinal infection, osteitis condensans ilii, severe or progressive scoliosis, spinal stenosis, spondylolisthesis, ankylosing spondylitis)
Complicated back problems (sciatica, back surgery in prior 3 years)
Other disabling chronic conditions that might confound treatment effects or interpretation of data (e.g., disabling heart or lung disease, diabetic neuropathy, active hepatitis, fibromyalgia, rheumatoid arthritis)
Acupuncture contraindicated or safety not confirmed (clotting disorders, on anticoagulant therapy, heart pacemaker, pregnancy, seizure disorder)
Medico-legal issues (seeking or receiving compensation/litigation for back pain)
Conditions possibly making consenting or treatment difficult (paralysis, inability to lie prone for 45 minutes, major psychoses, dementia, scheduling conflicts, severe vision or hearing problems, lack of transportation, unable to read or speak English)

### Recruitment procedures

Members of Group Health Cooperative in Seattle and Kaiser Permanente Northern California in Oakland whose visits to healthcare providers resulted in diagnoses consistent with non-specific low back pain will be identified from the health plans' automated visit data. Three to 12 months after their visit, potential participants will be mailed a letter that explains the study, describes eligibility requirements, and invites participation. Members interested in participating will sign and return a statement indicating their willingness to be contacted by study staff. An interviewer will then phone the members to answer questions and determine eligibility using a computer program to guide the members through a series of screening questions. If eligible, the study staff guides the patient through the consent process. Once written consent is obtained, an interviewer will contact the potential participant to administer the baseline questionnaire. If still willing to participate, participants will be randomized to one of the four groups. If the participant is randomized to acupuncture, the interviewer will schedule the first two acupuncture appointments.

If necessary, recruitment will be supplemented by advertising the study in the health plan's quarterly magazine, on its website, on bulletin boards in the health plans' medical centers, and by placement on the NIH clinical trials website (clinicaltrials.gov) Finally, if these methods fail to achieve recruitment goals, recruitment letters will be sent to randomly-selected health-plan members without a recent clinic visit for low back pain and who are not known to meet an exclusion criterion.

### Randomization to treatment groups

Separate randomization files will be created for each of the two sites. The random group assignments will be based on a block design where the block size is not constant.

At the end of each baseline interview, participants will be randomly assigned by computer to an acupuncture treatment or to continued access to usual care. At the time of randomization, the computer informs the Research Specialist and participant of assignment to an acupuncture treatment or to usual care. The specific acupuncture treatments participants are assigned to are only revealed to the treating acupuncturist, immediately prior to treatment. The random assignments cannot be viewed in advance and cannot be changed after randomization.

### Study treatments

In the three acupoint stimulation groups, participants will be treated twice weekly for the first three weeks, and then weekly for four weeks (a total of 10 treatments over seven weeks). Ten treatments were chosen because over 75% of the acupuncturists we surveyed believed 8 treatments would be necessary to treat chronic back pain [19]. These treatments will be provided at no cost to study participants. Electrostimulation, moxibustion, herbs, and other non-needle treatments and adjuncts will not be allowed in this trial.

All participants will be mailed the Back Pain Helpbook[20]within a week of randomization. This evidence-based manual for self-management of chronic back pain includes information on managing flare-ups, physical activity and exercise, and appropriate life-style modifications. From a practical viewpoint, we thought that giving this book to participants in the usual care group would minimize their disappointment at not receiving acupuncture treatment and would therefore reduce losses to follow-up. From a scientific viewpoint, providing the back-care book to all participants permits assessment of the efficacy of acupuncture beyond educational materials alone.

In our pilot study, we found that we could successfully mask participants to treatment group by using two acupuncturists who have minimal contact with each other: a "Diagnostician acupuncturist" who prescribes an individualized treatment plan at each visit and a "Therapist acupuncturist" who provides the treatment to which the participant was randomized. The Diagnostician acupuncturist will evaluate the participant at each visit using TCM diagnostic techniques and will then write a prescription for an individualized treatment designed to treat the back pain and any underlying TCM "constitutional deficiencies" that could prevent the pain from resolving or give rise to recurrences. A Research Specialist will then accompany the participant from the Diagnostician acupuncturist to the Therapist acupuncturist who will then administer the randomly assigned treatment, interacting minimally with the participant. Only one-third of participants randomized to an acupuncture treatment will actually receive the individualized treatment.

#### a) Individualized acupoint stimulation treatment

In this arm, the Therapist will administer the treatment prescribed at the beginning of each visit by the Diagnostician. This treatment may include acupoints on any part of the body that can be needled while the participant is lying prone with his/her head in a face cradle. There are no constraints on number of needles, depth of insertion, or needle manipulation. This arm of the trial resembles clinical practice where patients receive customized treatments that may vary from visit to visit.

#### b) Standardized acupoint stimulation treatment

We previously developed a standardized needling prescription (Table 2) considered effective for chronic low back pain [18]. All acupoints will be needled with sterile disposable 32-gauge needles (0.25-mm) for 20 minutes, with stimulation at 10 minutes and again just prior to needle removal. A 1.5-inch needle will be used for most acupoints, but longer needles will be used for acupoints where deeper needling is appropriate. Needles will be inserted to the depth typically recommended for that acupoint, generally between 1 and 3 cm [21]. Because 90% of the acupuncturists we surveyed felt that the phenomenon of *de qi *was important to successful treatment of back pain [19] it is an integral part of our treatment protocol. *De qi *is usually described by the patient as a feeling of numbness, heaviness or distention at the needle site [22]. However, there is also a biomechanical component to *de qi *that is felt by the practitioner. This "needle grasp response" occurs when tissue tightens around the inserted needle and constricts its movement [23], and is poetically described in the ancient texts as "the feeling of a fish biting on a fishing line" [24]. Therapists will detect *de qi *using the needle grasp response thereby avoiding discussion of the needling sensation with the patient and maintaining masking of the participant to treatment to the greatest extent possible. This approach will also be used in the individualized treatment.

**Table 2:** Standardized acupoint stimulation treatment

**Acupoints**	**Location**	**Major Indications and Actions**. [21,38]
Du3 (Yaoyang-guan)	Midline of the lower back, just below the spinous process of the fourth lumbar vertebra	Lumbosacral pain, motor impairment, numbness and pain of legs, other (e.g., seizures)
UB23 (Shenshu)	1.5 inches lateral to lower border of the spinous process of the second lumbar vertebra (bilateral point)	Low back pain, principal point to strengthen the "Kidneys", other (e.g., weakness of knee)
Low back ashi	Any tender point in the area between a horizontal line just below T12 and a horizontal line at the tip of the coccyx and extending to the outer contour of the body (as assessed by the Diagnostician acupuncturist)	Used according to the concept that "where there is pain, there is an acupuncture point"
UB40 (Weizhong)	Back of the knee in the midpoint of the transverse crease of the popliteal fossa (bilateral point)	Low back pain, motor impairment of hip joint, "activation of the UB channel"
Ki3 (Taixi)	On the foot, depression between the medial malleolus and tendo calcaneus (bilateral point)	Lumbar pain, strengthen lumbar spine, "nourishes Kidney Yin and tonifies Kidney Yang", other (e.g., insomnia)

#### c) Non-insertive acupoint stimulation treatment (simulated acupuncture)

We previously developed and tested a simulated acupuncture technique using a toothpick in a guidetube and determined that the technique was considered a credible acupuncture treatment by participants with low back pain [25] The acupuncturist will simulate insertion of needles, using the toothpick and guidetube technique, at the same eight acupoints used in the standardized treatment (Du3, UB23-bilateral, low back ashi, UB40-bilateral, Ki3-bilateral). Simulating insertion involves holding the skin taut around each acupoint and placing a standard acupuncture needle guidetube that contains a toothpick against the skin. The acupuncturist then taps the toothpick gently, twisting it slightly so that it feels to the participant like an acupuncture needle grabbing the skin, and then quickly withdraws both the toothpick and guidetube while keeping his or her fingers against the skin for a few additional seconds to imitate the process of inserting the needle to the proper depth. All acupoints will be "stimulated" with toothpicks at 10 minutes (i.e., the acupuncturist will touch each acupoint with the tip of a toothpick without the guidetube, rotate the toothpick clockwise and then counterclockwise less than 30 degrees) and again at 20 minutes just before they are "removed." Location of the correct acupoints for initial and subsequent stimulation will be facilitated by having marked all the acupoints with non-toxic ink prior to initiation of treatment. To simulate withdrawal of the needle, the acupuncturist tightly stretches the skin around each acupoint, presses a cotton ball firmly on the stretched skin, then momentarily touches the skin with a toothpick (without the guidetube) and quickly pulls the toothpick away using the same hand movements as in regular needle withdrawal. For verisimilitude, the Therapists will crinkle an empty needle wrapper to simulate the sound of unwrapping needles before treatment and will flick the side of the Sharps container after needle withdrawal to simulate the sound of needle disposal.

#### d) Usual care

We have included a usual care treatment arm to permit us to determine if individualized or standardized acupuncture offers advantages over standard care for chronic low back pain. Members of this group (as well as members of the three acupuncture treatment groups) will continue to receive the medical care they would have received in the absence of the study. This typically involves continued use of medications (mostly non-steroidal anti-inflammatory medicines), and occasionally, provider visits.

### Clinic sites

Acupuncture treatments will be performed in the Research Clinics at the two study sites. The Diagnosticians and Therapists will see participants in separate rooms. Therapists' rooms will resemble a typical acupuncture treatment room with a massage table for treatment and a table for acupuncture supplies. This table will be screened off from the massage table to prevent participants from looking at the needles or non-insertive implements to be used in their treatment.

### Study acupuncturists

Licensed acupuncturists will be recruited and trained as either Diagnosticians or Therapists and will function only in their assigned role throughout the study. All acupuncturists will be experienced TCM practitioners. We will require Diagnostician acupuncturists to have at least five years experience and Therapists to have at least three years experience with an emphasis on the treatment of musculoskeletal pain. All acupuncturists will need to agree to strictly adhere to the study protocol and to complete the rigorous training program developed in the pilot study. In addition, the Therapist acupuncturists will need to be comfortable administering all three treatments.

### Training and monitoring of acupuncturists and clinic staff

Prior to beginning the study, Acupuncturist Diagnosticians, Acupuncturist Therapists and Research Assistants will undergo intensive and customized training in the clinical protocol. There will be "dress rehearsals" with scripted "participants" to ensure that the clinical treatment team is able to adhere to the protocol under a variety of challenging participant scenarios. Much of the communication between the Therapists and the participants and between the Research Assistants and the participants is scripted and we have standard answers to commonly asked questions. The Research Assistants will use a special clinic visit form to document deviations from protocol by themselves or by the acupuncturists. Site visits will occur about 4 and 16 months after treatment begins to ensure continued adherence to study procedures.

### Assessment of outcomes

A core set of recommended outcome measures [26] covering five important domains will be assessed: back-related dysfunction, pain, general health status, disability and patient satisfaction. The primary efficacy/effectiveness outcomes are pain and dysfunction. There will be no physical assessments or laboratory tests because these have not been found to be useful for assessments of outcomes in studies of mechanical back pain. The primary assessment will be the 8-week telephone interview.

Table 3 below summarizes the categories of questions that are included in the baseline and follow-up questionnaires.

**Table 3:** Content of baseline and follow-up questionnaires

**Measures**	**Baseline**	**8-Week**	**26-Week**	**52-Week**
Sociodemographic characteristics	x			
Back pain history	x			
* **Roland Disability Questionnaire (dysfunction)**	x	x	x	x
* **Bothersomeness of low back pain**	x	x	x	x
Satisfaction with back care	x	x		x
General Health Status (SF-36)	x	x		x
Disability days	x	x	x	x
Medication use	x	x	x	x
Worry about back problem	x	x	x	x
Exercise (Back-related, general)	x	x		x
Confidence in ability to self-manage future back pain	x	x		x
Expectations of treatment	x			
Knowledge of acupuncture	x			
Adverse experiences		x		
Perceptions of acupuncture experience (acupoint stimulation participants only)		x		
Perceptions of book on self-care		x		x
Use of non-HMO services for back pain		x	x	x

Use and cost of HMO services for back pain [from automated HMO databases]

* Co-primary outcome measures

### Primary measures of efficacy/effectiveness

The modified Roland-Morris Disability Questionnaire will be used to measure back-related patient dysfunction [26,27]. This instrument, which asks 23 yes/no questions, takes approximately five minutes to complete. A composite score is obtained by summing up the number of "yes" responses, so the total scores range from 0 to 23. It has been found to be reliable, valid and sensitive to clinical changes [27-32] and is well suited for telephone administration.

Because there are individuals who are very bothered by even a small amount of pain and others who are not bothered by even moderate pain, our primary measure of symptoms assesses participants' perceptions of the impact of pain on their lives rather merely assigning a pain severity score. Thus, participants will be asked to rate how bothersome their low back pain has been during the past week on a 0 to 10 scale where 0 represents "not at all bothersome" and 10 "extremely bothersome". This measure appears to have substantial construct validity – i.e., it is highly correlated with measures of function and other outcome measures [27].

Both primary outcomes will be measured at baseline and during the 8, 26 and 52 week follow-up interviews. The trial's primary endpoint will be the 8 week follow-up, one week after completion of the 10 acupuncture treatments. All interviews will be conducted using computer-assisted telephone interviews (CATI).

### Secondary measures of efficacy/effectiveness

General health status will be measured using the well-validated SF- 36 [33] that has been recommended for use in studies of back pain [26,34]. The SF-36 measures eight health concepts: 1) limitations in physical activities because of health problems; 2) limitations in social activities because of physical or emotional problems; 3) limitations in usual role activities because of physical health problems; 4) bodily pain; 5) general mental health (psychological distress and well-being); 6) limitations in usual role activities because of emotional problems; 7) vitality (energy and fatigue); and 8) general health perceptions.

Satisfaction with information given about the cause of the back problem, treatments received, and overall care will be measured using three separate questions, each with a 5-point Likert scale (ranging from very satisfied to very dissatisfied). Days of restricted activity due to back problems, a surrogate for disability, will be measured with a back-pain specific modification of 3 National Health Interview Survey questions about the number of half-days spent in bed, home from work or school, or cutting down on usual activities due to illness or injury during the past week [35].

Efficacy will be determined by comparing outcomes among the four treatments arms, adjusting for baseline values. The analytic plan is described below.

### Measures of health care resource use

Participants' use of health care for back pain during the year following randomization will be determined from automated utilization data, interview data on out-of-plan utilization, and records for each visit to the study acupuncturists. The automated utilization data provide complete information about all provider visits and hospitalizations at health plan facilities or paid for by the health plan. Visits for back pain can be identified by the diagnosis listed for every visit and hospitalization. Imaging of the lower back can also be identified by specific procedure codes. Our previous studies have found that the automated data are as reliable as chart data for identifying provider visits and imaging for back pain, but much less costly.

Visits to non-study acupuncturists and providers not covered by the health plan will be estimated from the interview data for the time period since the previous interview. In other words, the 8-week interview will request information about out-of-plan utilization since participants were randomized to study treatment, the 26-week interview will ask about the previous 18 weeks, and so forth. To increase the accuracy of response, we will mail participants Health Care Utilization logs to track visits they make for their back pain shortly after randomization and after 8 and 26 weeks. This will permit determination of the percentage of participants in each treatment group who had any visits for low back pain during and following the intervention period, as well as estimation of the mean number of visits. Current use of medications of all types for low back pain will be captured in the follow-up interviews.

Costs of back-related care will be estimated by assigning dollar values to each service. Costs of specific services (e.g., visits, imaging studies, medications) will be determined using data from both sites' cost management information systems which define cost based on standard relative value units (RVUs) assigned to actual department RVUs produced. Costs of the acupuncture interventions will be distinguished from the costs of all other back pain-related services to permit separate comparisons among the treatment groups of the costs of the interventions themselves, of the non-intervention back care costs during the 7-week treatment period, and of the total back pain-related costs incurred over the 45 week period between completion of the study interventions and the end of the 52 week follow-up period.

## Protection of human subjects and assessment of safety

### Protection of human subjects

This study protocol was approved by the Institutional Review Boards of Group Health Cooperative and Kaiser Foundation Research Institute.

### Data safety monitoring board

The Data Safety Monitoring Board (DSMB) established by the National Center for Complementary and Alternative Medicine to monitor large trials will monitor the progress of the trial and review safety data.

### Adverse events

Participants will be asked about adverse experiences at each clinic visit and during the 8-week telephone interviews. We will define an adverse experience as any unfavorable and unintended sign, symptom or disease temporally associated with the use of the acupuncture treatments, which could reasonably be related to the procedure. Because acupuncture has relatively short-term physiological effects, we will not report adverse events that first manifest more than two weeks after a participant's final acupuncture treatment (or more than 9 weeks after randomization for the usual care control group). Any adverse event that is life-threatening or results in death, hospitalization, a persistent or significant disability/incapacity, a congenital anomaly/birth defect, or cancer will be promptly reported to the DSMB.

## Stopping rules

The trial will be stopped if the Data Safety Monitoring Board (DSMB) believes there is an unacceptable risk of serious adverse events in one or more of the treatment arms. No interim analyses of efficacy are planned to there are no formal stopping rules for efficacy or futility.

## Statistical issues

### Sample size and the detectable difference

The sample size was selected to have adequate power to detect a clinically significant difference of 2.0 points between groups on the Roland Disability scale. Our target sample size of 640 randomized individuals (160 per group) was computed using assumptions described below. Assuming 10% loss to follow-up for each group, there will be outcome data for 144 participants per group. The power of the overall analysis depends on how the four means are distributed. We used the pilot data to estimate the distribution of the means for the four groups and then computed an effect size that was used in the omnibus power calculation.

In the proposed analysis we will first determine if there is an overall difference among the four treatment groups using a two-sided significance level of 0.05. With 144 per group, this omnibus F-test will have 99% power for detecting a statistically significant difference among the four treatment means given the effect size obtained in the pilot study. If this omnibus test is statistically significant, we will compare specified pairs of means as indicated in the proposal. Because this will involve multiple post-hoc comparisons, we will use the Tukey-Kramer multiple comparisons procedure [36] to allow for all possible pairwise comparisons holding the overall α = 0.05. We will not consider a pairwise difference between the means to be statistically significant unless the t-value exceeds 2.61, instead of the usual 1.96.

Both primary outcomes (Roland Disability Questionnaire and bothersomeness of low back pain) are tested at the 0.05 level because they address separate scientific questions. The analyses of covariance of the 8-week pilot data provided estimates of the standard deviations of the primary outcome measures adjusted for pre-randomization baseline values: Roland SD = 4.71 and Bothersomeness SD = 2.73. Using these assumptions, the power to detect a pairwise difference of 2.0 Roland units is 84%, adjusted for multiple comparisons. For the bothersomeness outcome, for which a difference of 1.5 points is considered minimally clinically significant, we will have 98% power to detect a difference of this magnitude. Thus, if our assumptions are correct, 160 participants per group will provide adequate power to detect the smallest clinically meaningful differences on both of our primary outcome measures even after adjustment for multiple pairwise comparisons.

In our secondary analyses, we will compare the proportions of participants in each treatment group who experience clinically significant improvement on our primary outcome measures.

We plan to conduct subgroup analyses of gender, study site, and race/ethnicity that will include an overall comparison of the four groups followed by specific pairwise comparisons. Power for some of these comparisons will be limited. While we will test a site by treatment interaction to determine if the treatment effects differ across the two sites, this test will only be sensitive to very pronounced effect modification by site.

In summary, we have high power to find an overall difference among the four groups even in some of the planned subset analyses. Although powered to detect clinically significant difference on the Roland Disability scale, the resultant sample size will provide ample power for our other primary outcome, bothersomeness of back pain, even in pairwise comparisons adjusted for multiple comparisons. Because our estimates are conservative, we are very likely to have adequate power even if the assumptions on which our power estimates are based prove incorrect. In addition, the proposed sample sizes will permit us to explore whether the findings apply equally to women and men and to different racial groups.

### Statistical analysis

The power calculations are based on simple comparisons of the follow-up scores at a single point in time (eight weeks after randomization) with adjustment for baseline values using analysis of covariance. We also plan to adjust for other baseline characteristics. In a large randomized trial, baseline values and other covariates are unlikely to differ across randomized groups. However, for continuous outcome data, inclusion of baseline covariates can improve precision of the variance estimate and therefore increase power. The covariates to be used in the final model will be based on past experience and model fit as assessed prior to inclusion of treatment group in the model. We will use the Tukey-Kramer method to adjust for multiple pairwise comparisons [36] across the four groups and allow all pairwise comparisons to be conducted holding the overall α = 0.05 fixed.

We will use an intent-to-treat approach to all analyses, i.e. individuals will be analyzed by randomized group regardless of participation in any treatment sessions. This minimizes biases that often occur when participants not receiving assigned treatments are excluded from analyses. Furthermore, we will adjust for geographic site as a main effect. The standard linear regression model (ANCOVA) is of the form:

*Y*(*t*) = *β*_0 _+ *β*_1 _*Baseline *+ *α*_1 _Grp1+ + *α*_2 _Grp2 + *α*_3 _Grp3 + *θ*^*T *^*z *+ *ε*

where response is at follow-up time t, baseline is the pre-randomization value of the outcome measure, z is a vector of covariates representing other variables being adjusted for, including geographic site and, for some models, visits to various providers for back pain as part of usual care. The referent group is Usual Care in this model. We will consider interactions of treatment and the baseline value that would indicate that the effect of treatment depends on status at baseline. We will also test for significant interactions of treatment with other variables (e.g. site, race, gender) to determine if treatment differences are modified by these variables. The statistical model assumes that the error terms are normally distributed. In previous studies we have used transformations when this assumption was not satisfied. However, given that these patients have chronic back pain it is unlikely that most will return to a level of no pain or dysfunction so floor effects are not expected to be large. We will also investigate whether there are differences among the treating acupuncturists by inclusion of acupuncturist in the model as a random effect.

If we find a significant overall difference among the four groups for a particular outcome, we will localize the difference using pairwise comparisons in accordance with our specific aims (see Section 2 Project Overview). We will use the Tukey-Kramer procedure to adjust for multiple comparisons while maintaining an overall significance level of 0.05. Each specific aim will be addressed by a specific pairwise comparison.

Although this model evaluates differences at one time-point it can be extended to include all follow-up times to assess whether treatment effects change over time. This would be indicated by significant time by treatment interactions. For longitudinal analyses, we would use a generalized estimating equation (GEE) approach to protect against misspecification of the correlation within a participant's scores over time. Such analyses can incorporate every time point completed by a participant. However, it is important to assess whether there is differential drop-out by either assigned treatment or symptom or function level. If differential drop-out occurs, we will use the method of Little to rectify possible biases that could occur [37].

We will also analyze secondary outcomes: costs of back care, general health status (SF-36), pain intensity, disability days, and satisfaction with care. An analysis of costs may entail a transformation (e.g., log cost) but can be modeled using linear regression. The model is similar to the one described above except that we will not enter a baseline value for costs in the model since this is unknown. We will adjust for age and other factors that may affect costs. For binary outcomes (e.g., participants spending any days in bed due to their back pain during the past week) we will use logistic regression. Despite the multiplicity of analyses, we will focus our analyses on the effects of the treatments on each of the five outcome domains recommended for studies of low back pain [26].

## Competing interests

The author(s) declare that they have no competing interests.

## Authors' contributions

DC, KS, CH, WB, RD participated in the conception and design of the trial, in plans for the analysis of the data, and in drafting the manuscript. JE participated in the conception and design of the trial and in drafting the manuscript. AA participated in the design of the trial, in plans for the analysis of the data, and in drafting the manuscript. All authors read and approved the final manuscript.
